# TropicALL study: Thromboprophylaxis in Children treated for Acute Lymphoblastic Leukemia with Low-molecular-weight heparin: a multicenter randomized controlled trial

**DOI:** 10.1186/s12887-017-0877-x

**Published:** 2017-05-10

**Authors:** Irene L. M. Klaassen, Mandy N. Lauw, Marianne D. van de Wetering, Bart J. Biemond, Saskia Middeldorp, Floor C. H. Abbink, Marc Bierings, D. Maroeska M. W. te Loo, Rob Pieters, Inge M. van der Sluis, Wim J. E. Tissing, C. Michel Zwaan, C. Heleen van Ommen

**Affiliations:** 1 Department of Pediatric Hematology, Academic Medical Center, Amsterdam, The Netherlands; 2 Department of Vascular Medicine, Academic Medical Center, Amsterdam, The Netherlands; 3 Department of Hematology, Academic Medical Center, Amsterdam, The Netherlands; 4 Department of Pediatric Oncology, Academic Medical Center, Amsterdam, The Netherlands; 5 Department of Hematology/Oncology, VU Medical Center, Amsterdam, The Netherlands; 6 Department of Hematology/Oncology, Wilhelmina Children’s Hospital, Utrecht, The Netherlands; 7 Department of Pediatric Hematology/Oncology, Radboud University Medical Center, Nijmegen, The Netherlands; 8 Princess Máxima Center for Pediatric Oncology, Utrecht, The Netherlands; 9 Department of Pediatric Oncology/Hematology, Erasmus MC-Sophia Children’s Hospital, Rotterdam, The Netherlands; 10 Department of Pediatric Oncology, Beatrix Children’s Hospital, University Medical Center Groningen, Groningen, the Netherlands

**Keywords:** Acute lymphoblastic leukemia, Low molecular weight heparin, Venous thromboembolic disease, Asparaginase, Pediatric

## Abstract

**Background:**

Venous thromboembolism (VTE) is a common and severe complication during treatment of acute lymphoblastic leukemia (ALL). An important cause is the intensive use of asparaginase. Prospective cohort studies in which prophylactic low-molecular-weight heparin (LMWH) was used to prevent VTE showed lower VTE risk than in historic control cohorts, with a negligible bleeding risk. However, the efficacy of thromboprophylaxis with LMWH during ALL treatment has never been investigated in a randomized design. Here, we present the protocol of a randomized controlled trial in which the efficacy and safety of thromboprophylaxis with high prophylactic dose LMWH versus no thromboprophylaxis will be assessed in children treated for primary ALL with asparaginase.

**Methods/Design:**

Thromboprophylaxis in Children treated for Acute Lymphoblastic Leukemia with Low-molecular-weight heparin (TropicALL) is a multicenter, randomized controlled open-label trial conducted in the Netherlands. Patients between 1 and 19 years of age with primary ALL, who are treated within the Dutch Childhood Oncology Group (DCOG) ALL-11 or 12 study will be randomized to thromboprophylaxis with LMWH once daily, (dose of 85 IU/kg (intervention arm A)), or to no thromboprophylaxis (arm B, standard of care) during asparaginase courses of ALL treatment. Primary efficacy endpoint is symptomatic objectified VTE during ALL treatment; secondary efficacy endpoints are overall survival and the composite of symptomatic and asymptomatic objectified VTE. Primary safety endpoints are major bleeding, clinically relevant non-major bleeding and minor bleeding. A total of 324 patients will be included to obtain a relative risk reduction of 75% with a power of 80%, using a two-sided test with significance level α = 0.05.

**Discussion:**

This trial will be the first to assess efficacy and safety of thromboprophylaxis with LMWH during asparaginase treatment for ALL in children in a randomized design.

**Trail registration:**

Nederlands Trial Register NTR4707. Registered 30 July 2014.

## Background

Acute lymphoblastic leukemia (ALL) is the most common type of childhood cancer, representing a quarter of all pediatric malignancies [1, 2]. Survival rates for childhood ALL have improved significantly over the past decades, with a current 5-year survival of 86% [3, 4]. This is a result of substantial improvements in ALL treatment with risk-based therapy, increased treatment intensity and improved supportive care [5, 6].

Venous thromboembolism (VTE) is a frequent and severe complication during ALL treatment. Reported incidences of VTE during ALL treatment vary from 1 to 37% [7]. In unpublished data of the most recent Dutch Childhood Oncology Group (DCOG) ALL study, the ALL-10, incidence of symptomatic VTE was about 10% [3], but may have been underreported as VTE events were not systematically registered. The majority of VTE during ALL treatment are cerebral sinovenous thrombosis (CSVT) and catheter-related deep-vein thrombosis (DVT) (35–43%) [7–10]. CSVT can be severe and life-threatening [7, 8]. Reported overall mortality of CSVT varies between 10 and 21%, however limited data are available [7, 11]. Long term complications are common; more than 50% of patients with CSVT suffer from persistent neurological or cognitive impairments, and one fifth of patients has a reported poor quality of life [12–15]. Although mortality is rare in other types VTE such as (catheter-related) DVT, morbidity is frequent with for instance post-thrombotic syndrome (PTS), characterized by swelling, ulceration, pain and dilated collateral veins of the affected limb due to hampered venous flow, being frequently seen, with reported in up to 68% of patients [16–19].

Risk factors for VTE in ALL patients are well described but difficult to avoid. In particular, asparaginase therapy, which is an important component of ALL therapy, is considered to be a major risk factor for VTE as it reduces levels of natural anticoagulant proteins. Concomitant administration of corticosteroids may amplify this effect [7, 8, 20, 21]. In addition, a procoagulant state at diagnosis, presence of central venous catheters (CVCs), inherited prothrombotic defects and infections contribute to the risk of VTE [7, 8, 20, 22].

VTE occurrence leads to suboptimal treatment of ALL patients. In 68% of CSVT patients, therapy adjustments are necessary [11], which may lead to reduced survival rates [4]. In addition, CVC-related DVT is associated with recurrent catheter complications, such as obstruction of the catheter and catheter-related infections [23].

Currently, there are no evidence-based strategies to prevent VTE complications during ALL treatment. Administration of fresh frozen plasma (FFP) or antithrombin (AT) do not reduce the risk of VTE [24]. A few prospective cohort studies have been published which use low-molecular-weight heparin (LMWH) for thromboprophylaxis during ALL treatment. Elhasid et al. reported on 41 children with ALL who received LMWH prophylaxis during 4 to 8 courses in18–24 days of L-asparaginase treatment. During 76 courses of asparaginase, none of the children developed VTE and no bleeding episodes occurred [25]. Mitchell et al. described 19 children with ALL and increased risk for thrombosis, based on a VTE-risk score, including asparaginase, steroids, presence of CVC and thrombophilia. VTE developed in 1 of 8 children who were given prophylactic dose LMWH and in 8 of 11 children without thromboprophylaxis. No bleeding events occurred [26].

However, thromboprophylaxis with LMWH during ALL treatment has not been studied in a randomized design. As the prospective cohort studies showed promising results with a negligible bleeding risk, a large randomized controlled trial is needed to confirm these findings.

## Methods

### Aim and study design

The TropicALL study is a multicenter randomized controlled open-label trial conducted in the Netherlands. The aim of this study is to assess the efficacy and safety of thromboprophylaxis with LMWH in children treated for primary ALL in the DCOG ALL-11 or subsequent study. As a secondary objective, clinical risk factors will be evaluated to increase insight in the pathogenesis of VTE during ALL treatment and to establish a risk model for these complications. In addition, since a thrombophilic state is associated with a higher VTE risk, specific coagulation assays, including antithrombin, fibrinogen, D-dimer, thrombin antithrombin complex (TAT) and thrombin generation, and genetic thrombophilic mutations Factor V Leiden and prothrombin G20210A will be performed in patients with and without VTE. The inclusion period is 3 years with a follow-up of 3 months after the end of the ALL treatment period, or until recurrence of ALL, or until death. Primary efficacy endpoint is the incidence of symptomatic objectified VTE during ALL treatment; secondary efficacy endpoints are incidence of the composite of symptomatic and asymptomatic objectified VTE, and the value of plasma coagulation assays to predict the risk of VTE. Primary safety endpoint is major bleeding. Secondary safety endpoints are the incidence of clinically relevant non-major bleeding and minor bleeding, the burden of LMWH injections, and adverse skin reactions.

Patients will be closely monitored for adverse events during the study. All serious adverse events and unexpected adverse reactions, will be reported through the web portal ToetsingOnline to the accredited Ethics Committee that approved the study protocol. Furthermore a Data Monitoring Committee will semi-annually review all incidences of adverse events, symptomatic VTE and bleeding complications. To enhance safety of the study, DMC will provide the core committee with recommendations related to the protection of the patients’ safety, including stopping recruitment and study treatment.

### Definitions

Symptomatic VTE is defined as suspicion of VTE based on clinical symptoms, for instance for (CVC-associated) DVT: peripheral deep vein thrombosis, swelling, erythema, skin discoloration, increased warmth, pain, tenderness, venous distension, or presence of subcutaneous collateral veins and objectively confirmed by imaging tests. Other forms of VTE (i.e. pulmonary emboli and CSVT) are defined as symptomatic venous thrombosis in any component of the venous or pulmonary arterial circulations or the heart, or the cerebral sinovenous system, requiring therapeutic anticoagulation, acute intervention, life-saving measures, ALL treatment adjustments, or of fatal nature, and objectively confirmed by routine imaging tests. Screening for asymptomatic VTE should not be performed. However, if thrombi are incidentally found upon diagnostic imaging for other indications, these are counted as asymptomatic VTE. Treatment of these asymptomatic VTE will be determined by the attending physician.

Bleeding is categorized as major, clinically relevant non-major or minor bleeding according to Perinatal and Pediatric Subcommittee of the Scientific and Standardization Committee of the International Society on Thrombosis and Haemostasis criteria [27]. A bleeding is considered as major if it is a fatal bleeding, clinically overt bleeding associated with a decrease in hemoglobin of at least 3.1 mmol/l in a 24-h period, bleeding that is retroperitoneal, pulmonary, intracranial, or otherwise involves the central nervous system; and bleeding that requires surgical intervention in an operating suite. A clinical relevant non-major bleeding is defined as an overt bleeding for which a blood product is administered and not directly attributable to the patient’s underlying medical condition or a bleeding that requires medical or surgical intervention to restore hemostasis, not in an operating suite. All other overt bleedings or macroscopic evidence of bleeding are considered minor.

Burden of LMWH injections is estimated by a visual analogue scale (VAS) score and defined as a VAS score > 5.

### Study population

Children between 1 and 19 years of age with primary ALL, who are treated within the DCOG ALL-11 or subsequent study, are eligible for the TropicALL. Patients will be excluded if they are already using anticoagulant therapy upon screening, if they have active bleeding or a high risk for bleeding contraindicating anticoagulant therapy, including renal insufficiency (glomerular filtration rate (GFR) < 30 ml/min/1.73 m2), hepatic disease associated with coagulopathy leading to a clinically relevant bleeding risk, stage 2 hypertension defined as blood pressure confirmed >99th percentile +5 mmHg, if they have a heparin allergy or a history of heparin-induced thrombocytopenia (HIT), or any other condition that, judged by the investigator, would place the patient at increased risk of harm due to participation. Thrombocytopenia is no exclusion criteria. However, LWMH should be temporarily interrupted, if platelet levels drop below 20 × 10^9^/L.

### ALL-11 study design

**Fig. 1:**
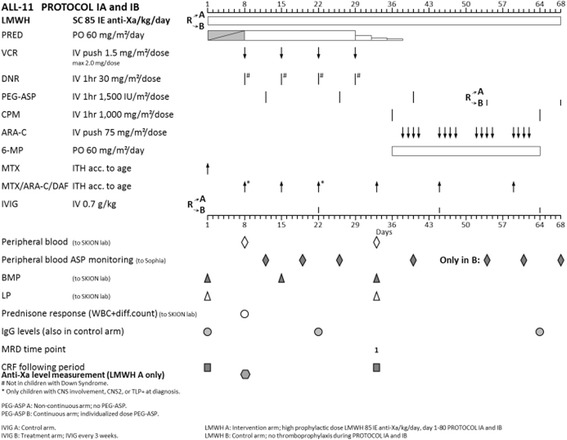
LMWH randomization in DCOG Protocol ALL 11, protocol 1A and 1B

**Fig. 2:**
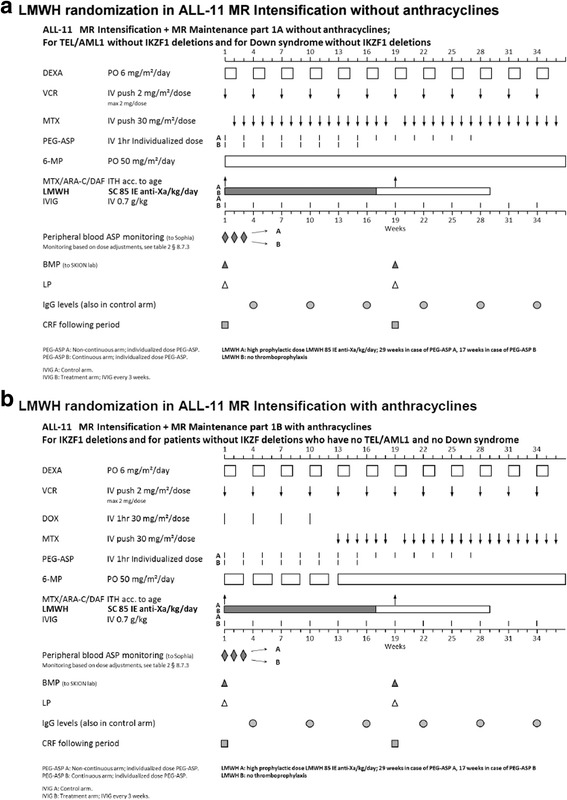
**a** LMWH randomization in DCOG Protocol ALL 11, MR intensification, without anthracyclines. **b** LMWH randomization in DCOG Protocol ALL 11, MR intensification, with anthracyclines

All patients in ALL-11 are treated with a standardized treatment schedule. (Figs. 1, 2a and b.) All patients start with Induction IA and IB, after which patients are stratified according to their risk group. After risk group stratification, patients with Standard Risk ALL will be treated with cycle M, followed by cycle IV and maintenance therapy. Medium Risk group patients will be treated with cycle M, followed by Medium Risk Intensification and maintenance cycles. High Risk group patients will be treated with cycle M if identified by minimal residual disease (MRD) or with High Risk blocks if identified earlier. After three High Risk blocks, patients will undergo assessment for allogeneic stem cell transplantation eligibility, or will continue High Risk blocks. PEG-asparaginase is included in Induction IA (day 12, 26, 4; 1500 IU/m2) and, in case of continuous asparaginase treatment, IB (day 54, 68; individualized dose) and M (day 9, 23, 37, 51; individualized dose). In Medium Risk group patients (70% of all patients), PEG-asparaginase is administered every 2 weeks for 14 times or 8 times during Intensification, depending on the randomization between standard and continuous asparaginase treatment. Both arms have the same number of total PEG-asparaginase administrations.

### Intervention; LMWH regimen

LMWH (nadroparin) will be given subcutaneously once daily, in a standardized prophylactic weight- and anti-Xa adjusted dose of 85 IU/kg with a maximum of 5700 IU/day [28]. Previous studies have indicated that especially young children require higher dosages of LMWH. Hence, peak anti-Xa levels should be measured, 3 to 5 days after start of LMWH prophylaxis, with an aimed anti-Xa level of 0.3–0.4 IU/ml [29]. Lidocain/prilocain cream can be applied to the skin locally for anesthesia before each subcutaneous injection. If nadroparin is not tolerated, other LMWHs can be administered according to local availability (Table 1). A registration booklet and registration of unused drugs will be used to monitor treatment compliance. The booklets will also include questions on the burden of daily subcutaneous injections.

**Table 1:** Alternative LMWH schedule

LMWH	Dose
Enoxaparin	1 – 18y: 1 mg/kg once daily s.c
Dalteparin	65 ± 21.5 IE/kg once daily s.c
Tinzaparin	1 – 5y: 120 IE/kg once daily s.c.
	5 – 10y: 100 IE/kg once daily s.c.
	10 - 18y: 87.5 IE/kg once daily s.c.

### Data and blood sample collection

At the time of randomization for the TropicALL study and during the study, data will be collected from all patients, including baseline data, clinical risk factors for VTE, CVC characteristics, number of episodes of septicemia, asparaginase therapy and administration of other medications during ALL treatment.

Upon inclusion, one EDTA blood sample will be collected for evaluation of genetic thrombophilic mutations Factor V Leiden and Factor II mutation. Blood samples for coagulation assays, including antithrombin, fibrinogen, D-dimer, thrombin antithrombin complex (TAT) and thrombin generation, will be collected upon, during the induction cycle before the 2nd and 3rd asparaginase administration, and in Medium Risk group children in week 1 of Intensification and on the last day of PEG-asparaginase therapy of Maintenance. All coagulation assays will be performed at the end of the study. Blood collection dates coincide with other set sample collection dates in ALL-11 or subsequent study and will not form an extra burden for patients.

### Sample size calculation

Power calculations were based on an estimated symptomatic VTE incidence of 10% based on the previous DCOG ALL-10 study, and an estimated relative risk reduction (RRR) of 75% based on the abovementioned cohort studies with LMWH in childhood ALL [25, 26, 30, 31]. Using nQuery Advisor (7.0, 2007), it was calculated that 162 patients are required in each arm for a power of 80%, using a two-sided test with significance level α = 0.05. Hence, 324 patients will be randomized in this study.

### Randomization

Patients will be randomized to thromboprophylaxis with LMWH (arm A) or to no thromboprophylaxis (arm B, standard of care). Randomization will take place on day 11, which is the last day before the start of PEG-asparaginase administration during the induction cycle. A register of all patients, who were eligible but not included in the study, will be kept and reason for refusal of participation will be recorded. Randomization of each patient will be performed by a randomization computer program at the DCOG trial office and will be stratified to ensure equal distribution of patients over study arms. Stratification will be done according to type of ALL (B-cell or T-cell) and study center. The procedure will also guarantee concealment of allocation by the treating physician.

### Treatment arms in TropicALL

All patients randomized to arm A will receive LMWH from the day asparaginase is started, until 28 days (for PEG-asparaginase) after the end of asparaginase therapy, in all treatment cycles with asparaginase (Induction cycle, Intensification in Medium Risk protocol). In case PEG-asparaginase is replaced by Erwinia asparaginase, LMWH will be given until 7 days after the last administration of Erwinia asparaginase. Patients treated according the medium risk arm of the protocol will receive 34 weeks (=238 days) of LMWH in the induction and intensification cycles, while patients treated according to the standard risk and high risk ALL-11 protocols will receive LMWH in the induction cycle only (=42 days during and after asparaginase). Patients in arm B, the standard of care arm, will not receive any form of thromboprophylaxis.

LMWH should be stopped 24 h before interventions, i.e. lumbar puncture or CVC insertion, and should be restarted after invasive procedures or surgical interventions within 24 h, provided the clinical situation allows. Moreover, LMWH should be temporarily interrupted if platelet levels drop below 20 × 10^9^/L, and in case of major bleeding.

### Safety measures

If major bleeding occurs, the following measures should be considered: (1) delay the next LMWH administration or discontinue treatment, (2) consider protamine sulfate administration, (3) consider usual treatment for bleeding, including blood transfusion, and/or fresh frozen plasma, (4) measure anti-Xa level of LMWH. If bleeding cannot be controlled, consider administration of recombinant factor VII (Novoseven®) or prothrombin complex concentrate.

### Reporting of symptomatic VTE events

Patients are monitored for clinical symptoms of VTE during all treatment cycles of ALL-11 and a 3-month follow-up period. To establish systematic registration of symptomatic VTE, events must be prospectively centrally recorded on special VTE reporting forms in the CRF. VTE reporting forms include several specifications of the VTE to enable complete analysis of the event and its consequences for the patient and ALL treatment. For each symptomatic, objectively diagnosed VTE event in a study patient, a VTE reporting form should be completed and returned to the DCOG Office within 14 days after the event. Recurrent VTE should also be recorded.

Presence, replacement, removal or reinsertion of a CVC should be documented in the CRF, as well as the applied method of catheter flushing.

### Statistical analysis

All data will be analyzed according to the intention-to-treat principle. Descriptive data will be presented as mean with its corresponding standard deviation if normally distributed, and medians with ranges if data are skewed. Depending on the data distribution the Student’s *t* test or a Mann-Whitney U-test will be used to compare continuous variables between the treatment groups. Categorical variables will be analyzed using the Chi-square test.

Efficacy and safety of LMWH to prevent VTE will be analyzed by the difference between the incidence proportions and cumulative incidences of the primary efficacy and safety outcomes, symptomatic objectified VTE and major or clinically relevant non-major bleeding, respectively, observed from randomization up to 14 and 7 days after the last PEG asparaginase and Erwinia asparaginase, respectively. A two-sided test with significance level α = 0.05 will be used.

All bleeding events that occur during LMWH or within 2 days after cessation of LMWH will be included. Incidence proportions (number of children with outcome during the period divided by number of children at risk at the beginning of the period) and cumulative incidences will be estimated by a joint model to estimate event free survival for the primary safety outcome. To identify which risks factors are associated with time to VTE a joint model for longitudinal data and survival outcome will be used.

### Ethical considerations

The study has been approved by the Ethics Committee of the Erasmus Medical Center in Rotterdam, the Netherlands, and the local Ethics Committee of each participating hospital. Patients can only be included in this study after obtaining written informed consent of both parents and children aged 12 and older. Verbal and written information on all parts of the study should be given.

We acknowledge, daily subcutaneous injections for the entire duration of asparaginase therapy could be a burden for patients. However, the high risk of VTE and potentially severe complications deriving from this VTE, transcend potential burden of dialy injections. Lidocain/prilocain cream will be applied to diminish the pain of subcutaneous injections. To evaluate the burden, patients will receive a booklet with questions.

### Withdrawal of patients

Children can discontinue their participation in the study at any time for any reason if they wish to do so without any consequences for their care, at their own request or at the request of their parents/legally acceptable representative. Furthermore, study medication can be stopped prematurely in case of serious adverse events, VTE requiring therapeutic anticoagulant treatment, or if, in the investigator’s opinion, study medication should be stopped for any other reason.

## Discussion

Despite the fact that thromboprophylaxis with LMWH has been proven to be safe in children for the prevention of VTE, there are no randomized studies that have investigated the use of thromboprophylaxis with LMWH during ALL treatment [32]. The TropicALL will be the first randomized controlled trial to investigate the safety and efficacy of LMWH as thromboprophylaxis in children treated for primary ALL. As a result of its incorporation in the DCOG ALL-11 and subsequent study protocols, optimal enrolment of patients is secured as well as implementation in all involved pediatric oncology centers. With the results of this study, we intend to establish (inter)national guidelines with recommendations for thromboprophylaxis during childhood ALL treatment.

## Abbreviations

ALL: Acute lymphoblastic leukemia
AT: Antithrombin
CVC: Central venous catheter
CSVT: Cerebral sinovenous thrombosis
DCOG: Dutch Childhood Oncology Group
DVT: Deep venous thrombosis
FFP: Fresh frozen plasma
GFR: Glomerular filtration rate
HIT: Heparin-induced thrombocytopenia
LMWH: Low-molecular-weight heparin
PTS: Post thrombotic syndrome
RRR: Relative risk reduction
VTE: Venous thromboembolism
